# Determination of Crack Tip Plastic Zone Using Dynamically Visible Mechanochromic Luminescence Response

**DOI:** 10.3390/ma18081810

**Published:** 2025-04-15

**Authors:** Yuhan Tong, Yonggang Ren, Zhe Zhang

**Affiliations:** 1 School of Chemical Engineering and Technology, Tianjin University, Tianjin 300350, China; coolper1215881648@tju.edu.cn (Y.T.); ryg3018001587@tju.edu.cn (Y.R.); 2 Zhejiang Institute, Tianjin University, Ningbo 315201, China; 3 Tianjin Key Laboratory of Chemical Process Safety and Equipment Technology, Tianjin 300350, China

**Keywords:** crack tip plastic zone, failure analysis, mechanochromic luminescence, image processing, correlation analysis

## Abstract

The plastic zone shields the crack tip from high stress and plays an important role in the fracture of structures. Determination of the plastic zone is a significant challenge in large-scale and complex structures. In the present work, a detection method using mechanochromic luminescent (MCL) sensing film has been proposed to detect the plastic zone near the crack tip. The deformation near the crack tip is converted into visible green fluorescence emission. A comprehensive post-processing protocol and a feature quantification scheme for fluorescence images are introduced. A significant correlation is obtained between the characteristic values of fluorescence images and the parameters of the plastic zone (i.e., maximum equivalent strain and plastic zone size), indicating that the fluorescence response provides effective characterization parameters within the forward model. The plastic zone parameters determined using the MRL-based method agree well with the results measured using the DIC method. This indicates that the plastic zone near the crack tip can be effectively analyzed by capturing loading conditions and fluorescence response.

## 1. Introduction

Structural defects in aircraft and nuclear power pipelines inevitably generate local damage during service, potentially resulting in catastrophic consequences [1,2]. Consequently, monitoring the initiation and propagation of the damage is imperative for evaluating structural safety. The plastic zone shields the crack tip from high stress and plays an important role in the fracture of structures [3,4,5]. However, the plastic zone depends on many factors, such as the mechanical properties of the material, the state of stress, the governing size, and so on [6,7,8]. Thus, the measurement of the plastic zone near the crack tip is important for evaluating the crack growth and fracture toughness. There are many methods that have been used to detect the plastic zone, such as the microhardness method [9], etching method [10], optical interference method [11], foil strain-gauge method [12], and digital image correlation (DIC) method [13]. Nevertheless, the aforementioned methods are inconvenient and expensive for the measurement in large-scale and complex structures.

Mechanoresponsive luminescent (MRL) materials can convert external force to light emission or color alternation, which provides a new idea for structural damage detection [14,15]. In general, the MRL-based sensing film is produced and coated on the structural surface. Then, the MRL response of the sensing film is triggered by structural damage, which is also used to evaluate the structural integrity [16,17,18]. The mechanoluminescent (ML) phenomenon has been confirmed in various inorganic materials, including alkali halides [19,20,21], SrAl_2_O_4_ [22,23,24], ZnS [25,26,27], and SrAMgSi_2_O_7_ [28], which have been employed in numerous studies for real-time visualization of local strain and crack propagation. However, the reported ML materials encounter a number of challenges due the poor film-forming capability and interface debonding. Moreover, the instantaneous ML signals also bring inconvenience to data collection and transmission. In comparison, mechanochromic luminescent (MCL) materials exhibit a much longer luminescence response under other light excitation, thereby providing more convenience for structural damage detection and data transmission [29]. In recent years, organic MCL molecules with aggregation-induced emission (AIE) characteristics have obtained widespread attention due to their great film-forming capability and high force sensitivity [30]. It has been reported that 1,1,2,2-tetrakis (4-nitrophenyl) ethene (TPE-4N) sensing film has been used successfully to monitor the stress/strain distribution and fatigue crack propagation in structures [31,32,33,34]. Furthermore, the MCL response can be used to evaluate the fatigue crack length and fatigue life [35,36].

However, current analyses of MCL responses predominantly focus on qualitatively demonstrating that fluorescence signals can identify damaged areas and indicate damage severity. These investigations remain relatively superficial, as the inability to quantify fluorescence signal features hinders the transformation of MCL responses into precise numerical parameters such as the stress/strain distribution. Especially, the local deformation-induced fluorescence response in the plastic zone near the crack tip is weak. Namely, the low fluorescence intensity and non-uniform distribution cause a great deal of trouble for quantitative analysis of the stress/strain distribution, which also brings many difficulties in analyzing the size and shape of the plastic zone. In the study of the plastic zone, the maximum equivalent strain at the crack tip and the plastic zone size often serve as two critical parameters [37]. Therefore, it is essential to quantify the features of fluorescence images using the image analysis method and to establish the relationships between the effective characteristic values of fluorescence images and the parameters of the plastic zone. For the extraction of fluorescence image characteristic values, Wei et al. employed a global fixed threshold method to extract regions of local strain concentration from fluorescence images, subsequently calculating the average grayscale value as the fluorescence intensity [35,36]. Nevertheless, this method is highly subjective and does not adapt well to fluorescence images with grayscale histograms that lack clear double peaks [38]. Therefore, the present work employs a watershed image segmentation algorithm grounded in topological theory to accurately segment the fluorescent area with blurred boundaries near the crack tip. The watershed algorithm conceptualizes the image as a topological landform, where the gray value of each pixel corresponds to its elevation [39]. The segmentation process objectively preserves the details of the strain-concentrated region, enabling the extraction of more accurate simple characteristic values for the target area [40].

The forward model effectively elucidates the quantitative relationship between various non-destructive testing (NDT) signals and structural damage [41]. Establishing a robust forward model necessitates the extraction of effective characterization parameters from the NDT signals, as well as the verification of their intrinsic mapping relationship with the stress/strain distribution at defects. Wu et al. extracted magnetic-flux-leakage (MFL) gradient vectors from biaxial three-dimensional MFL signals generated during quasi-static tensile tests on both defect-free and defective wide-plate tensile specimens [42]. A correlation between the magnitudes of MFL gradient vectors and the equivalent stress distribution vertical to the loading direction obtained by the finite element method (FEM) and DIC was confirmed. Additionally, five important characteristic values were extracted to quantitatively assess the degree of stress inhomogeneity at the defect. As mentioned above, the low intensity and inhomogeneous distribution of fluorescence response generate difficulty in calibration between strain and fluorescence response. Therefore, the proposed work investigates the determination methods for the plastic zone near the crack tip using the dynamically visible MRL-based detection method. The local deformation near the crack tip is detected using the visible MCL response of the TPE-4N sensing film. A variety of computer vision methods are employed to extract characteristic values from fluorescence images, primarily including feature quantification methods based on enhanced watershed segmentation, grayscale histograms, and Gray Level Co-occurrence Matrix (GLCM). Meanwhile, the DIC measurement is applied to determine the maximum equivalent strain at the crack tip and the plastic zone size. Then, the extracted characteristic values were validated as effective characterization parameters for fluorescence signals through Spearman correlation analysis. Finally, a specific mapping relationship between the characteristic values of crack-tip fluorescence images and two parameters about the plastic zone was established using multiple linear regression, thereby completing the construction of the comprehensive forward model. The present work provides a new method for dynamically visible detection and evaluation of fracture damage.

## 2. Materials and Methods

**Figure 1:**
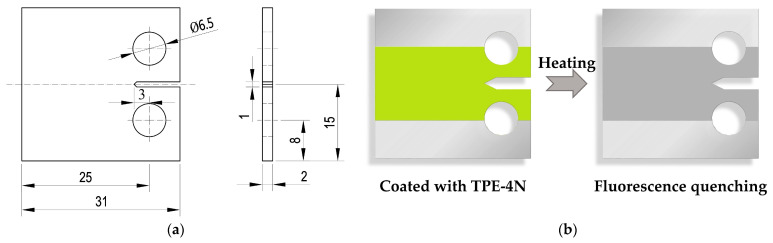

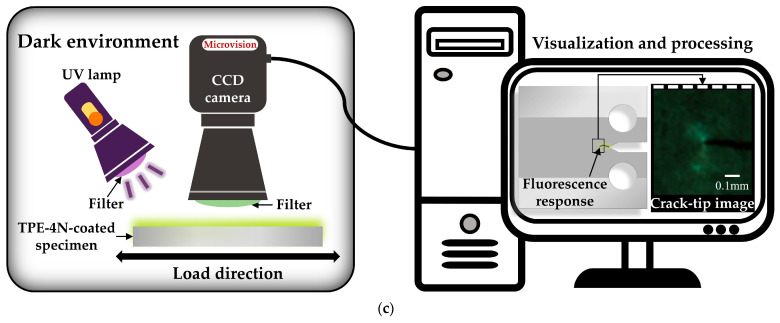
(**a**) Shape and dimension of the CT specimen; (**b**) Preparation of the TPE-4N-coated specimen; (**c**) schematic of the experimental setup.

In this study, a 2 mm thick 2024-T4 aluminum alloy substrate was used. Compact tensile (CT) specimens were strictly prepared following ASTM E1820 standards, as shown in Figure 1a. The specimens have a width (*W*) of 25 mm, a thickness (*B*) of 2 mm, and an initial notch length (*a*_0_) of 3 mm. According to the ratio of the crack length to the width (*a*/*W*), after machining openings of 1 mm, 6 mm, and 11 mm at the initial crack gap in three specimens using a wire-cutting machine, a 1 mm crack was prefabricated with an electro-hydraulic servo fatigue testing machine (MTS Bionix 370.2). Therefore, the CT specimens with *a*/*W* values of 0.2, 0.4, and 0.6 were prepared, respectively. The precracking was performed under load control at a frequency of 2 Hz with a stress ratio of 0.1.

The preparation of the CT specimen coated with TPE-4N sensing film is illustrated in Figure 1b. A TPE-4N/chloroform solution was prepared at a concentration of 0.02 g/mL and coated on the surface of the specimen using a drop-coating process. At this time, an amorphous TPE-4N film was formed on the surfaces of the specimen, emitting green fluorescence under UV light. Subsequently, the specimen was heated to 150 °C for 15 min, resulting in the transformation of the TPE-4N film into a crystalline state, accompanied by quenching of the green fluorescence. After that, the specimen was deformed under uniaxial loading; then, the fluorescence appeared near the crack tip.

The schematic of the experimental setup is shown in Figure 1c, which includes two parts: the mechanical testing and fluorescence signal acquisition system, as well as the fluorescence signal visualization and processing system. The tensile experiment was conducted in a dark environment to eliminate the influence of external ambient light. The MTS system was utilized to conduct uniaxial tensile tension with a load rate of 9 N/s. The fluorescence signal acquisition system comprised a charge-coupled device (CCD) camera equipped with a telephoto lens, a 10-watt 365 nm ultraviolet (UV) lamp, a light-emitting diode (LED) lamp, and two filters. The CCD camera and lens, manufactured by Microvision, model MV-EM510M/C, feature a maximum frame rate of 15 fps, a resolution of width 2456 × height 2058 pixels, and an aperture of f/8. The image acquisition interval during stretching was controlled at 1 s. A total of 366 sets of fluorescence images near the crack tip from CT specimens with three different *a*/*W* values were captured during the experiment. For the post-processing of fluorescence images, ImageJ 1.54 was employed to register the series of fluorescence images. Subsequently, computer vision libraries such as OpenCV and Scikit-image, based on Python 3.11, were utilized to carry out the remaining image processing steps and extract characteristic values of fluorescence images. In order to obtain the features of the plastic zone, the DIC method was used to measure the maximum equivalent strain at the crack tip and the plastic zone size. The software Ncorr 2D v1.2 in MATLAB R2021b was utilized to analyze the DIC results. Ultimately, Statistical Package for the Social Sciences (SPSS) 28.0 was employed to investigate the correlation between the characteristic values of fluorescence images and the plastic zone parameters near the crack tip. Concurrently, specific mapping relationships were established by SPSS, linking the characteristic values to both the maximum equivalent strain and the plastic zone size.

## 3. Results

### 3.1. Mechanoresponsive Fluorescence Response near the Crack Tip

**Figure 2:**
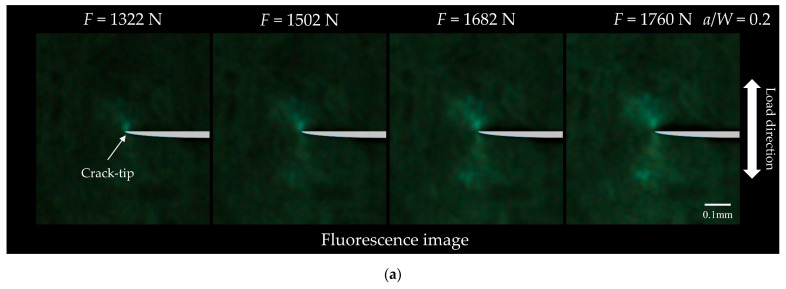

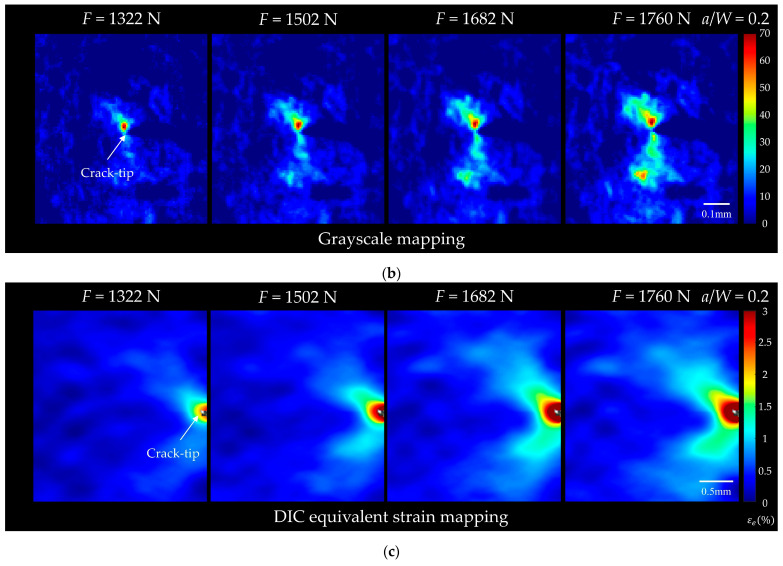
Representative fluorescence responses of TPE-4N film on the surface of the CT specimen near the crack tip under uniaxial tension (*a*/*W* = 0.2): (**a**) fluorescence image; (**b**) grayscale mapping; (**c**) corresponding equivalent strain distribution using DIC method.

Figure 2a illustrates the typical mechanoresponsive fluorescence responses near the crack tip of the CT specimen with *a*/*W* = 0.2 under the vertical uniaxial tensile loading (width 100 × height 110 pixels). It is seen that the visible green fluorescence emits near the crack tip, indicating that the local deformation near the crack tip is converted into the visible mechanoresponsive fluorescence of TPE-4N film. To eliminate the uncertainty in the overall brightness of the fluorescence images caused by environmental factors or the experimental setup, the images presented in Figure 2a were normalized by subtracting the corresponding background brightness from the grayscale images of fluorescence images. For enhanced visualization, the grayscale images are represented as pseudo-color mappings in Figure 2b. Figure 2c presents the equivalent strain distribution near the crack tip obtained using the DIC method under the same condition. It is interesting to note that the fluorescence distribution obtained using the proposed MRL method agrees well with the equivalent strain distribution measured using the conventional DIC method. It is explained that the local strain near the crack tip of the substrate generates the change of crystalline structure of the surface TPE-4N film, resulting in fluorescence emission [30]. Therefore, the visible fluorescence response is utilized to estimate the maximum equivalent strain at the crack tip and the plastic zone size.

However, the changes observed in the grayscale values of the fluorescence images are significantly less sensitive compared to the variations in the equivalent strain measured by the DIC method. As the applied load increases, a greater number of pixel points with grayscale values exceeding 30 appear in the fluorescence image. However, the maximum grayscale value consistently remained around 70. This observation suggests that the fluorescent area at the crack tip gradually expands, but the maximum fluorescence intensity remains relatively stable. Conversely, in the equivalent strain mapping measured by DIC, the equivalent strain does not exceed 2% at *F* = 1322 N, whereas the equivalent strain in the vicinity of the crack tip approached 3% at a load of 1760 N. Additionally, the fluorescence distribution near the crack tip is far less uniform than the equivalent strain distribution observed through the DIC method. The aforementioned problems bring many difficulties in accurately recognizing the size and shape of the plastic zone. Therefore, image processing is performed, and the features of fluorescence images are quantified to evaluate the maximum equivalent strain and the plastic zone size in the present work.

### 3.2. Fluorescence Image Processing

**Figure 3:**
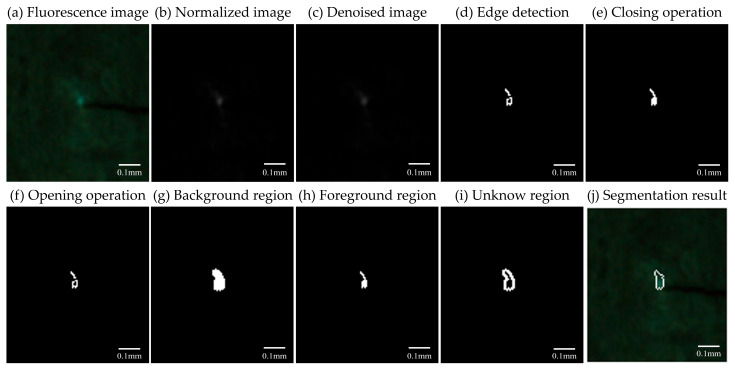
Typical image processing analysis of the mechanoresponsive fluorescence image (*a*/*W* = 0.2, *F* = 1322 N).

Figure 3 illustrates the image processing of the obtained fluorescence images. As indicated in Figure 3b,c, image pretreatment is carried out. It is found that the crack position in each fluorescence image captured by the CCD camera may be slightly displaced due to the movement of the specimen during uniaxial tensile tests. Consequently, an initial image is selected as the reference, and the subsequent images are translated and rotated to ensure that the cracks consistently appear in the same location utilizing the StackReg rigid registration model plugin in ImageJ. The selected fluorescence image near the crack tip (width 100 × height 110 pixels) is cropped from each original image. Firstly, background brightness interference is eliminated, resulting in a normalized grayscale image composed of relative grayscale values. Then, the application of a Gaussian linear spatial filter to smooth and suppress noise in the normalized image.

Applying threshold-based image segmentation algorithms presents many challenges due to the absence of significant double peaks in the grayscale histogram of fluorescence images. To effectively segment the continuously varying fluorescent area near the crack tip (i.e., the region of interest (ROI)), an enhanced watershed image segmentation algorithm that combines edge detection and morphological processing is proposed. The Canny edge detector, derived from a second-order function, transcends basic template convolution techniques and identifies edge points using gradient direction and dual-threshold strategy [43,44]. Figure 3d displays the binary image obtained after segmenting the fluorescent area near the crack tip using the Canny operator. The inherently high degree of blurriness at the edge of the fluorescent area near the crack tip makes it challenging to accurately detect a precise and closed edge using solely the edge detection operator. Thus, morphological processes are further applied to the result of edge detection. Figure 3e illustrates the result obtained after applying a closing operation to the detected edge. It is evident that the closing operation effectively fills the interior of the edge. However, closing operation cannot produce a smooth delineation of the fluorescent area edge. Figure 3f illustrates the result of an opening operation applied to the result of edge detection. The irregular noise can be effectively removed. To achieve superior segmentation results, an expansion operation is initially applied to the opening operation. This process aims to obtain a large background region that encompasses the entire fluorescent area along with a portion of the background (see Figure 3g). The next step involves utilizing the distance transform function to calculate the Euclidean distance from any white point in the closing binary image to the nearest background point. This process is equivalent to determining the distance between non-zero pixels and the closest zero-value pixel, resulting in a conservative foreground region that certainly encompasses the ROI (see Figure 3h). The distance transform is applied to the result of the closed operation rather than the opening operation. It is seen from Figure 3i that subtracting the foreground region from the background region results in an unknown region, including both the irrelevant background and the ROI. A connected component is defined as a collection of pixels in an image that possess identical properties and are interconnected. By analyzing these connected components, it becomes feasible to identify watersheds where dams may need to be constructed within the context of the watershed image segmentation algorithm. Therefore, by comparing the labeled connected components in two binary images (i.e., Figure 3h,i), the continuously varying fluorescent area near the crack tip can be effectively segmented (see Figure 3j).

**Figure 4:**
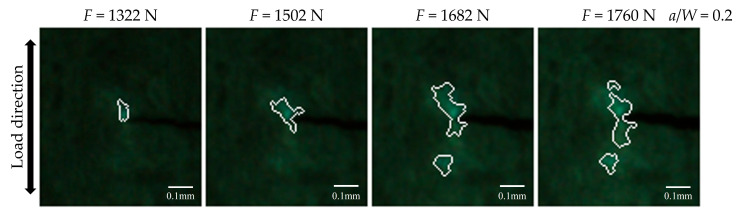
Representative results of segmenting fluorescent area of the CT specimen crack tip (ROI) under different tensile loads.

The remaining representative fluorescence images in Figure 2a, captured under various vertical tensile loadings, are segmented ROI utilizing the enhanced watershed image segmentation algorithm described above, as illustrated in Figure 4. The fluorescent area at the crack tip has been successfully segmented, and the size of the ROI gradually increases as the load increases. However, the segmented fluorescent area does not exhibit complete symmetry with respect to the crack propagation direction, and there are instances where a part of the ROI is not effectively segmented. This limitation arises from the challenge faced by edge detection in accurately capturing the regions where the fluorescence response is relatively weak, i.e., the contrast with the image background is insufficient.

## 4. Discussion

### 4.1. Fluorescence Image Features Quantification

The information derived from an image is referred to as image features, which facilitates the accurate distinction of each image from other images. The primary features of an image include its shape, color, texture, and spatial connection features, among other factors. To obtain an accurate numerical solution for the plastic zone parameters near the crack tip, it is essential to extract as many effective characteristic values from the fluorescence image as possible to quantify image features. In the proposed work, three distinct types of characteristic values are extracted to investigate the features of fluorescence images.

#### 4.1.1. Simple Characteristic Values Extraction

**Figure 5:**
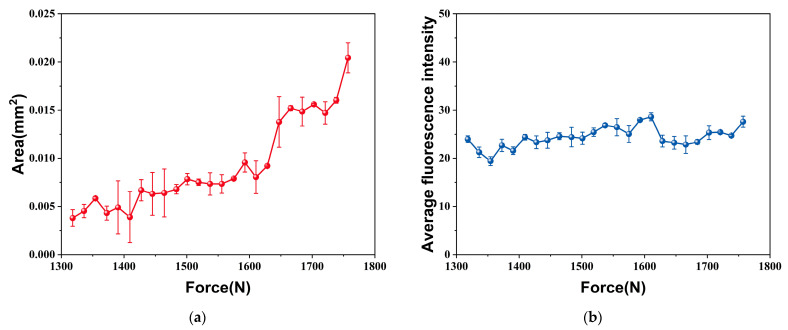
Evolution of the simple characteristic values under uniaxial tension (*a*/*W* = 0.2): (**a**) Area; (**b**) Average fluorescence intensity.

As indicated in Figure 4, the ROI increases gradually with the increasing tensile load. Therefore, both the area and average fluorescence intensity of the ROI can be extracted as simple characteristic values, which visually represent the variation in the fluorescence response near the crack tip. As shown in Figure 5, the evolution tendencies of the simple characteristic values in response to the applied load are characterized by the arithmetic means of five independently replicated experiments. Overall, it is seen from Figure 5a that the increased applied load induces an increase in the ROI area, but the area exhibits some fluctuations with increasing applied load. By contrast, it is seen from Figure 5b that the change in the average fluorescence intensity in the ROI is not significant with the increasing applied load. This phenomenon can be attributed to the fact that the rate of increase in average fluorescence intensity within the ROI is considerably less pronounced than that in area.

#### 4.1.2. First-Order Characteristic Values Extraction

The grayscale histogram of an image serves as a robust tool for characterizing the distribution of pixel grayscale levels. Given that the grayscale levels distribution in textured regions exhibits a distinct pattern, first-order characteristic values can be extracted from the grayscale histogram of the fluorescence image to elucidate its texture features.

Various first-order characteristic values are calculated using the grayscale histogram of the normalized, denoised grayscale image of the fluorescence image near the crack tip. These values include the mean value, standard deviation, smoothness, uniformity, entropy, and skewness. The mean value (*m*) serves to quantify the average brightness of the image texture, as demonstrated in Equation (1):

(1) $$m=\sum_{i=0}^{L-1}z_{i}P(z_{i})=\frac{\sum_{i=0}^{L-1}z_{i}h(z_{i})}{\sum_{i=0}^{L-1}h(z_{i})},$$

wherein, *L* is the total number of grayscales in the image, *z_i_* is the *i*-th grayscale, *P*(*z_i_*) is the probability with the grayscale of *z_i_* in the normalized grayscale histogram, and *h*(*z*_i_) is the number of pixel points with the grayscale of *z_i_* in the unnormalized grayscale histogram. The standard deviation (*σ*) quantifies the average contrast of the image texture, as indicated in Equation (2):

(2) $$\sigma =\sqrt{\sum_{i=0}^{L-1}{{(z}_{i}-m)}^{2}P(z_{i})}.$$

The smoothness (*R*) is utilized to evaluate the relative smoothness or roughness of texture brightness, as shown in Equation (3):

(3) $$R=\frac{1}{\left(1+\sigma^{2}\right)}.$$

A smoothness value close to one is assigned to areas with high consistency of grayscale, while a value close to zero is assigned to areas exhibiting significant variations of grayscale. The uniformity (*U*) reflects the degree of similarity among the grayscale values within the texture region, as presented in Equation (4):

(4) $$U=\sum_{i=0}^{L-1}{P\left(z_{i}\right)}^{2},$$

and it reaches its maximum when all grayscale values in the region are identical. The entropy (*e*) quantifies randomness, as shown in Equation (5):

(5) $$e=-\sum_{i=0}^{L-1}{P(z_{i})}^{2}{log}_{2}P(z_{i}).$$

A higher entropy value reflects greater randomness and information content, while a lower entropy value suggests increased certainty and diminished information content. The skewness (*μ*_3_) measures the symmetry of the grayscale distribution, as illustrated in Equation (6):

(6) $$\mu_{3}=\sum_{i=0}^{L-1}{{(z}_{i}-m)}^{3}P(z_{i}).$$

In a perfectly symmetric grayscale histogram, the skewness equals zero. A positive value signifies a right skew, whereas a negative value indicates a left skew.

**Figure 6:**
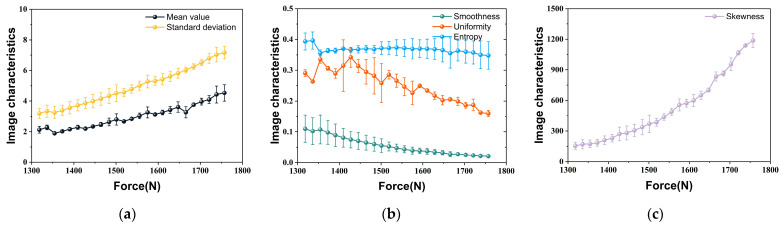
Evolution of first-order characteristic values under uniaxial tension (*a*/*W* = 0.2): (**a**) Mean value and standard deviation; (**b**) Smoothness, uniformity, and entropy; (**c**) Skewness.

Figure 6 illustrates the variation in the arithmetic means of the first-order characteristic values with the increasing applied load derived from the five experiments. The average brightness and contrast of the fluorescence image texture consistently increase throughout the experimental process, which generates the increasing mean value and standard deviation. Interestingly, a phenomenon has been observed wherein the standard deviation exceeds the mean value. This occurrence can be attributed to the fact that the majority of pixels in the grayscale mappings of crack-tip fluorescence images are extremely dark, while a small subset of pixels exhibits extreme brightness. Consequently, the high-intensity pixels deviate from the mean to a significantly greater extent than the mean itself. The results of smoothness, uniformity, and entropy decreased during the experiment. The smoothness of significantly less than one indicates a considerable disparity in the grayscale values of the fluorescence image, attributable to background brightness elimination. The introduction of new grayscale levels further contributes to the ongoing decrease in smoothness and uniformity, ultimately leading to smoothness approaching zero. The reduction in entropy indicates a decrease in the amount of information present within fluorescence images. Moreover, the skewness remains consistently greater than zero and continues to grow, indicating that the grayscale histogram of the fluorescence image is skewed to the right, with the degree of skewness increasing rapidly. The underlying cause of this phenomenon is attributed to the increased intensity of the fluorescence response at the crack tip, which leads to the development of more new pixel points with high gray values, thereby rapidly extending the right tail of the grayscale histogram.

#### 4.1.3. Second-Order Characteristic Values Extraction

First-order characteristic values have a limited capacity to describe the texture of an image, thus they may not adequately capture the spatial relationship between pixels or information about the boundary structure of objects. Additionally, they are not robust to noise. These limitations are effectively addressed by second-order characteristic values extracted from the Gray Level Co-occurrence Matrix (GLCM) of an image. The GLCM is a two-dimensional data structure that characterizes the joint distribution of pairs of pixels exhibiting a specific spatial positional relationship.

Let *f*(*x*, *y*) be a grayscale image of size *M* × *N*, and the number of grayscales is *K*. Then the GLCM of this image can be represented by Equation (7):

(7) $$P\left(i,j\right)=\{\left(x_{1},y_{1}\right),(x_{2},y_{2})\in M\times N|f\left(x_{1},y_{1}\right)=i,f\left(x_{2},y_{2}\right)=j\},$$

with matrix size *K* × *K*. Where *i* and *j* represent two grayscale values, (*x*_1_, *y*_1_) and (*x*_2_, *y*_2_) are pixels in the image. If the distance between these two pixels is defined as *d* and the calculation direction as *θ*, then GLCM *P*(*i*, *j*, *d*, *θ*) for the specified distance and direction can be obtained.

Haralick identified 14 s-order characteristic values that can be derived from the GLCM [45]. The most prominent of these characteristic values include contrast, dissimilarity, homogeneity, Angular Second Moment (ASM), energy, and correlation. Contrast can reflect both the clarity of the texture and the depth of the furrows, as indicated in Equation (8):

(8) $$Contrast=\sum_{i=0}^{K-1}\sum_{j=0}^{K-1}{\left(i-j\right)}^{2}P(i,j),$$

wherein, *P*(*i*, *j*) is a (normalized) frequency of occurrence of grayscale value pair (*i*, *j*). Weights are grown exponentially with the distance of matrix elements from the diagonal to calculate the contrast. If the weight is altered to linear growth, the dissimilarity is obtained, as calculated in Equation (9):

(9) $$Dissimilarity=\sum_{i=0}^{K-1}\sum_{j=0}^{K-1}\left|i-j\right|P(i,j).$$

Equation (10):

(10) $$Homogeneity=\sum_{i=0}^{K-1}\sum_{j=0}^{K-1}\frac{P(i,j)}{1+{\left(i-j\right)}^{2}},$$

can be used to determine the local homogeneity of the image texture. Equation (11):

(11) $$ASM=\sum_{i=0}^{K-1}\sum_{j=0}^{K-1}{P(i,j)}^{2},$$

and Equation (12):

(12) $$Energy=\sqrt{\sum_{i=0}^{K-1}\sum_{j=0}^{K-1}{P(i,j)}^{2}},$$

demonstrate how the ASM and energy are computed to represent the uniformity of the grayscale distribution and the coarseness of the texture, respectively. Equation (13):

(13) $$Correlation=\sum_{i=0}^{K-1}\sum_{j=0}^{K-1}\frac{\left(i-\mu_{x})(j-\mu_{y})P(i,j\right)}{\sigma_{x}\sigma_{y}},$$

illustrates the linear relationship between a pixel and its neighboring pixels, serving as a measure of local grayscale correlation in the image. Wherein, *μ_x_*, *μ_y_*, *σ_x_*, *σ_y_* are the mean and variance of the row and column sums, respectively, defined as Equations (14)–(17):

(14) $$\mu_{x}=\sum_{i=0}^{K-1}\sum_{j=0}^{K-1}iP(i,j),$$

(15) $$\mu_{y}=\sum_{i=0}^{K-1}\sum_{j=0}^{K-1}jP(i,j),$$

(16) $$\sigma_{x}=\sqrt{\sum_{i=0}^{K-1}\sum_{j=0}^{K-1}{\left(i-\mu_{x}\right)}^{2}P(i,j)},$$

(17) $$\sigma_{y}=\sqrt{\sum_{i=0}^{K-1}\sum_{j=0}^{K-1}{\left(j-\mu_{x}\right)}^{2}P(i,j).}$$

**Figure 7:**
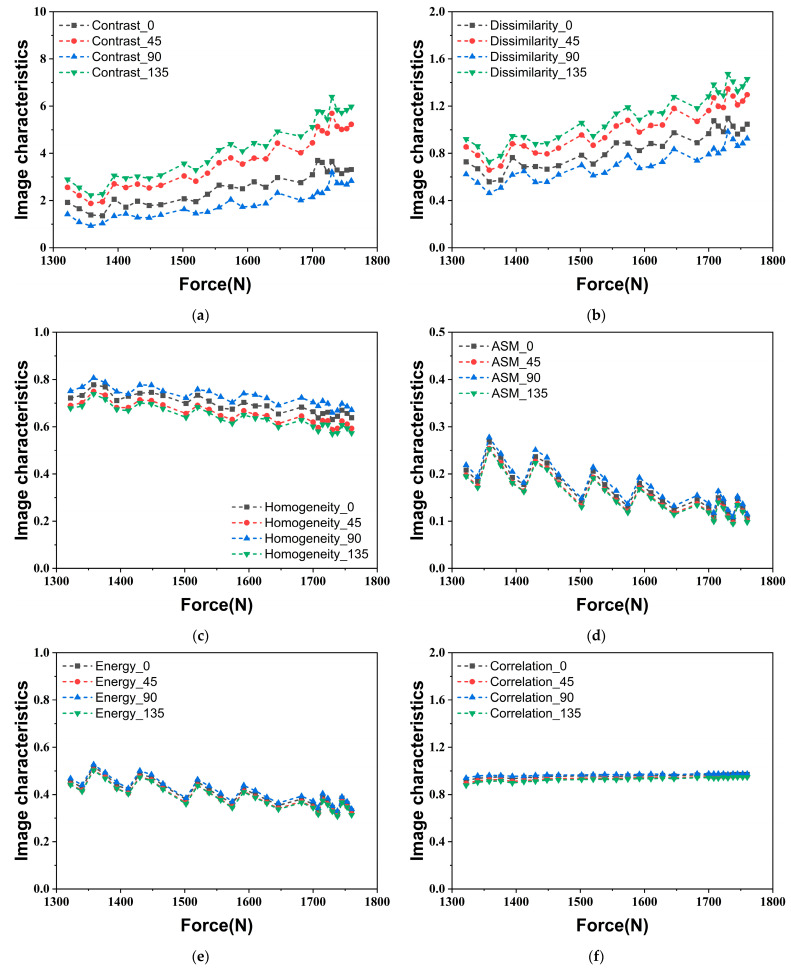
Evolution of second-order characteristic values in four directions under uniaxial tension (*a*/*W* = 0.2): (**a**) Contrast; (**b**) Dissimilarity; (**c**) Homogeneity; (**d**) ASM; (**e**) Energy; (**f**) Correlation.

**Figure 8:**
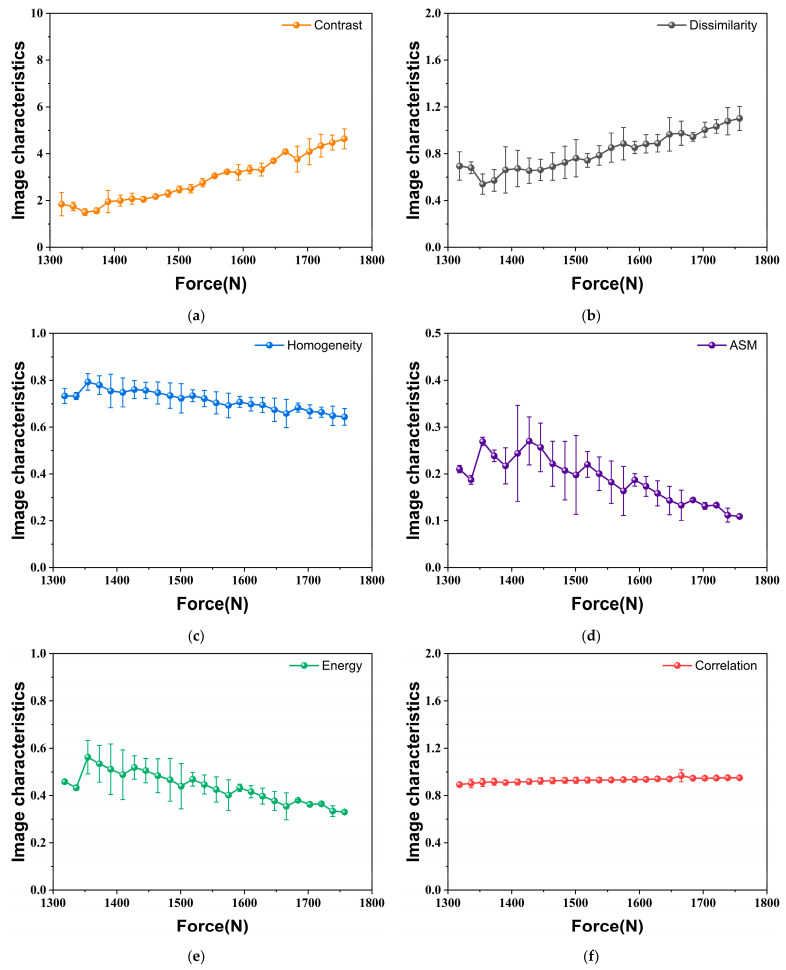
Evolution of second-order characteristic values averaged over four directions under uniaxial tension (*a*/*W* = 0.2): (**a**) Contrast; (**b**) Dissimilarity; (**c**) Homogeneity; (**d**) ASM; (**e**) Energy; (**f**) Correlation.

In this study, the distance *d* is set to 1 due to the small size and delicate texture of the fluorescence images at the crack tip. Subsequently, the angle *θ* is set to 0, 45°, 90°, and 135°, allowing for the acquisition of the corresponding GLCM of the normalized, denoised grayscale image of the fluorescence image near the crack tip by scanning from each of these four directions. Each fluorescence image yields 24 s-order GLCM characteristic values, which are plotted in the line charts as shown in Figure 7, illustrating their variation with the applied load. For the homogeneity, ASM, energy, and correlation, the results obtained from the four directions show almost no difference; in particular, the four curves of ASM, energy, and correlation essentially overlap completely. In contrast, calculating from different directions, the values of contrast and dissimilarity demonstrate relatively distinct differences. The values at the loading direction (*θ* = 90°) and the horizontal direction (*θ* = 0) are significantly lower than those observed in the diagonal directions. The minimum values are found in the loading direction, while the maximum values are noted in the direction where the fluorescence response at the crack tip expands (*θ* = 135°). The phenomenon indicates that these two characteristic values can detect texture changes in different directions of the fluorescence images during crack propagation, demonstrating the strong sensitivity of the partial second-order characteristic values obtained by GLCM to small morphological changes of the sensing film on the specimen surface. Although there are variations in the absolute magnitudes of these six characteristic values across different directions, the overall trend of variation remains consistent. As uniaxial loading increases, the levels of contrast and dissimilarity rise, indicating that the clarity of texture and the depth of the furrows in the fluorescence image gradually improve. Conversely, the values of homogeneity, ASM, and energy decline, suggesting that the grayscale distribution becomes more inconsistent. Moreover, the correlation shows little variation, revealing that the degree of similarity between the grayscale of the fluorescence image in the row and column directions remains stable. In summary, to evaluate the second-order GLCM characteristic values of a fluorescence image more comprehensively, the average value across the four calculation directions is selected as the second-order characteristic values of the fluorescence image. Synthesizing the results of the five experiments, Figure 8 presents the variation in the average values of the second-order characteristic values calculated from the four directions under different applied loads. Notably, the error bands for the ASM and energy are longer than those for the remaining four second-order characteristic values, suggesting that they exhibit less stability.

### 4.2. Plastic Zone Determination Using DIC Method

**Figure 9:**
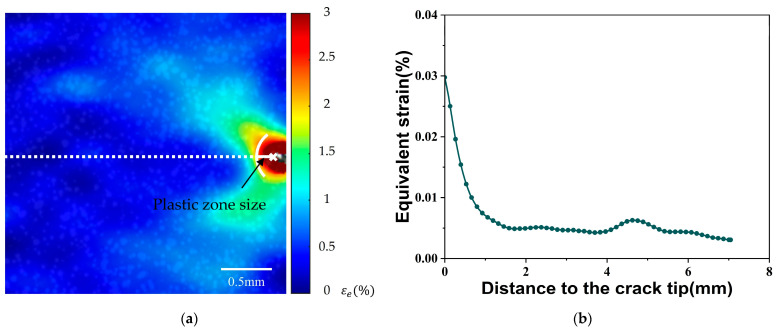
(**a**) Typical equivalent strain field measured by DIC (*a*/*W* = 0.2, *F* = 1760 N); (**b**) equivalent strain along the white dotted line.

To present the maximum equivalent strain at the crack tip and the plastic zone size using the characteristic values of the fluorescence image and experimental conditions, the DIC method was used to determine the experimental results of two plastic zone parameters under the same condition. Figure 9a illustrates the typical equivalent strain distribution and plastic zone near the crack tip obtained using the DIC method. Figure 9b depicts the decay of equivalent strain from the crack tip marker along the white dot line. The maximum equivalent strain occurs at the position closest to the crack tip along the horizontal white dotted line. As reported, the plastic zone is defined as the region where the strain exceeds 2% [46]. The distance between the 2% strain boundary and the crack tip is regarded as the plastic zone size along the crack plane [47].

**Figure 10:**
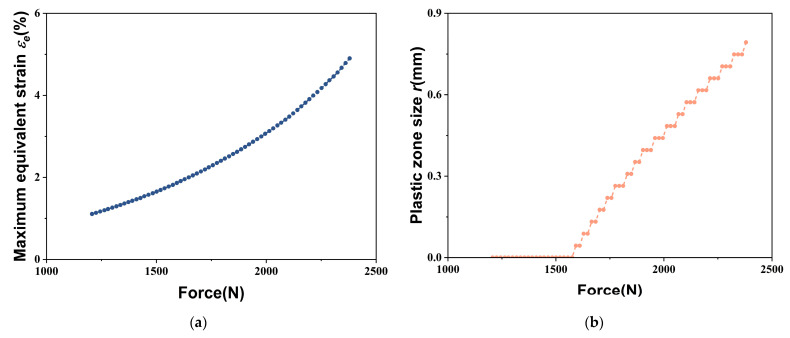
(**a**) Variation of maximum equivalent strain; (**b**) Variation of plastic zone size.

The change of the maximum equivalent strain at the crack tip throughout the uniaxial tensile deformation is shown in Figure 10a. It is observed that the maximum equivalent strain gradually increases with increasing applied load. Figure 10b plots the variation of the plastic zone size near the crack tip under the different applied loads. None of the equivalent strain values in the vicinity of the crack tip exceeded 2% for relatively low tensile loads, in which case the size of the plastic zone can be defined as zero. The plastic zone develops near the crack tip when the tensile load increases to approximately 1600 N. Subsequently, the plastic zone size exhibits an approximately linear increase with further increased applied load.

### 4.3. Correlation Analysis Method Investigation

**Figure 11:**
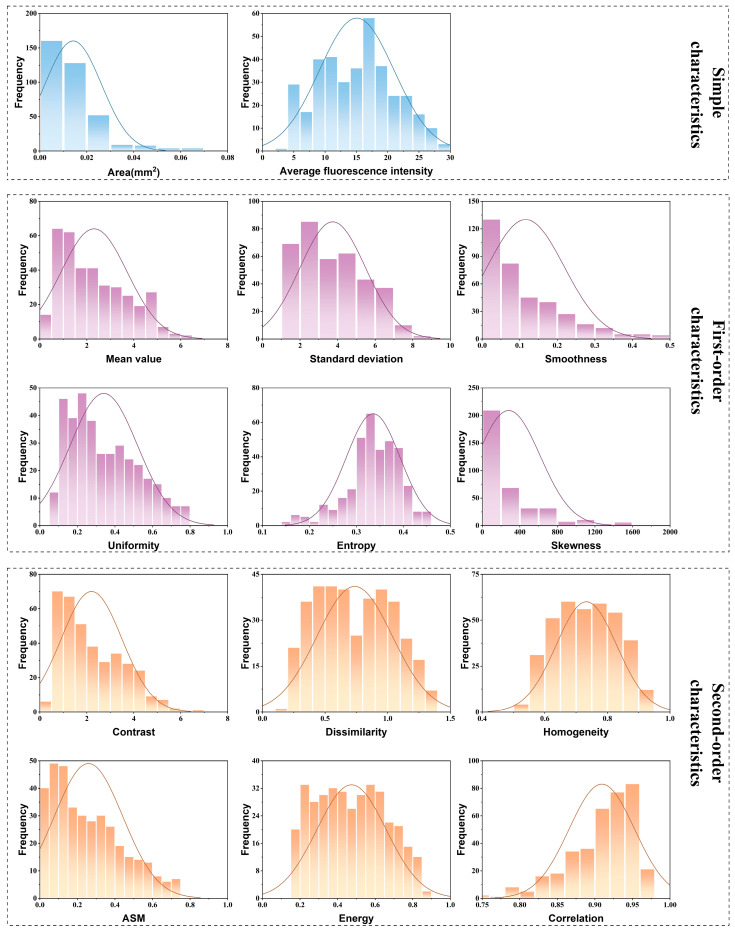
Histogram normality test for characteristic values of the obtained fluorescence images.

The most effective and precise statistical technique for examining the correlation between continuous numerical variables is Pearson linear correlation analysis. However, this method requires that the variables under examination must adhere to a normal distribution. Figure 11 discusses the histograms of two simple characteristic values, six first-order characteristic values, and six second-order characteristic values obtained from all experiments, along with their corresponding normal curves. All 14 characteristic values of the fluorescence image display a distinctly skewed distribution. The Kolmogorov–Smirnov (K-S) test and Shapiro–Wilk (S-W) test, both of which utilize nonparametric methods for normality testing, were employed to further validate the results of the normality assessment as shown in Table A1, Table A2 and Table A3. The results of the histogram method described above are supported by the fact that both nonparametric testing methods rejected the null hypothesis that the characteristic values are normally distributed. In summary, the Pearson linear correlation analysis method is not suitable for conducting correlation analysis between the characteristic values of the fluorescence image and plastic zone parameters. Therefore, it is essential to employ Spearman rank correlation analysis, a nonparametric statistical technique that assesses correlation by examining the ranks of data objects. The Spearman rank correlation analysis can be applied to both ordered categorical variables and continuous numerical variables that do not obey normal distribution.

The Spearman rank correlation coefficients between the 14 characteristic values of the crack-tip fluorescence image and the maximum equivalent strain at the crack tip and the plastic zone size were calculated separately. The resulting 28 correlation coefficients are presented in Table 1, Table 2 and Table 3. All correlation coefficients are marked with two asterisks, signifying that the *p*-values are all less than 0.01. This indicates that all characteristic values of the fluorescence image exhibit very significant correlations with the maximum equivalent strain at the crack tip and the plastic zone size. When examining the correlation coefficient, a value greater than zero indicates a significant positive correlation between the two variables, whereas a value less than zero signifies a significant negative correlation. Furthermore, if the absolute value of the correlation coefficient exceeds 0.4, it denotes a strong and significant correlation between the two variables; otherwise, it reflects a weak and significant correlation.

The area of the ROI demonstrates a significant and strong positive correlation with both the maximum equivalent strain and the plastic zone size. The average fluorescence intensity within the ROI exhibits a significant and strong positive correlation with the maximum equivalent strain at the crack tip, while it shows only a significant but weak positive correlation with the plastic zone size. Regarding first-order characteristic values, the mean, standard deviation, and skewness are significantly and strongly positively correlated with both the maximum equivalent strain and the plastic zone size. Conversely, smoothness and uniformity display significant and strong negative correlations with the two parameters related to the plastic zone, while entropy reveals a significant but weak negative correlation. Among the second-order characteristic values, contrast, dissimilarity, and correlation present significant and strong positive correlations with the two parameters for assessing the plastic zone. In contrast, homogeneity, ASM, and energy manifest significant and strong negative correlations with the plastic zone parameters.

### 4.4. Determination of Plastic Zone Parameters

The results of the Spearman rank correlation analysis indicate a highly significant correlation between all 14 characteristic values of the deformation-induced fluorescence images and the two plastic zone parameters, i.e., the maximum equivalent strain at the crack tip and the plastic zone size. Moreover, the correlation coefficients demonstrate a distinct linear relationship between standard deviation and smoothness, as well as between ASM and energy. Consequently, among the four characteristic values, only standard deviation and ASM are chosen as the effective characterization parameters for further analysis. At the same time, to establish a high-precision forward model that relates quantified features of fluorescence images to the plastic zone parameters, the tensile load *F* and *a*/*W* value are also used as the variables. The numerical mapping between 14 explanatory variables (i.e., 12 characteristic values of the fluorescence image near the crack tip and two experimental variables) and two response variables (i.e., maximum equivalent strain at the crack tip and plastic zone size) is ultimately fitted using multiple linear regression (MLR) methods. However, if the experimental environment, specimen material, or other conditions change, the mapping relationship established in this study will also be affected. Therefore, the specific multiple linear regression formula is not provided here; instead, the focus is on analyzing its assessment accuracy.

Table 4 presents the four evaluation indicators of the multiple linear regression fitting. Two coefficients of determination (R^2^) concerning the maximum equivalent strain at the crack tip and the plastic zone size are 0.91568 and 0.86262, respectively. Both values exceed 0.8, indicating a highly accurate assessment. The mean absolute error (MAE), mean square error (MSE), and root-mean-square error (RMSE) are all approximately zero, suggesting a negligible difference between the assessed and experimental values. In particular, the calculation of the MAE reveals that the estimation error for the maximum equivalent strain is approximately 0.00227, while for the plastic zone size, it is around 0.04986. This implies that the estimation error can be effectively confined within the range of adjacent captured fluorescence images.

**Figure 12:**
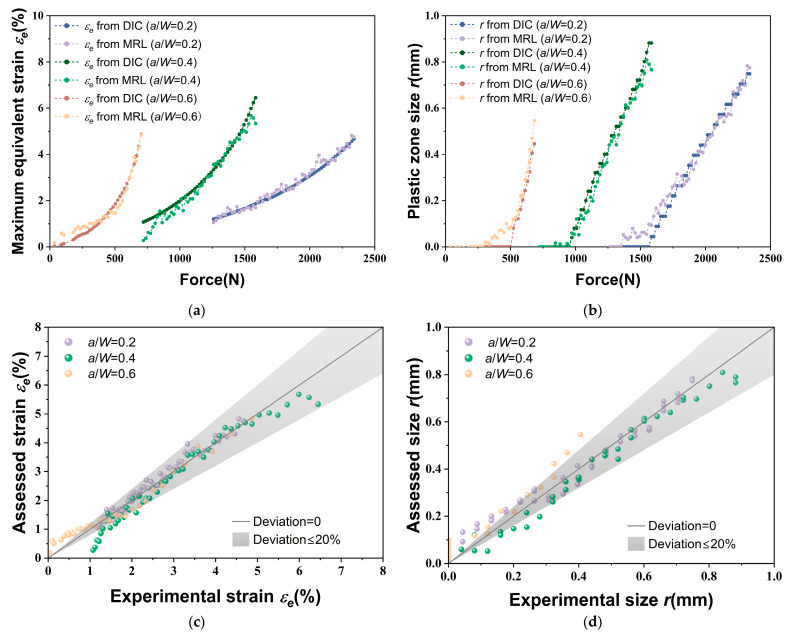
Comparison of the maximum equivalent strain and the plastic zone size between DIC experimental results and values obtained from the MRL-based method: (**a**) Maximum equivalent strain across various loads; (**b**) Plastic zone size across various loads; (**c**) Maximum equivalent strain at different *a*/*W*; (**d**) Plastic zone size at different *a*/*W*.

Figure 12 presents a comparison of the maximum equivalent strain and the plastic zone size between the DIC experimental results and the values obtained from the MRL-based method. As shown in Figure 12a,b, the experimental results exhibit a strong alignment with the values derived from the MRL-based method under various tensile loads. However, at low applied loads, the fluorescence response resulting from the strain concentration is also minimal. This significantly complicates the extraction of characteristic values from the fluorescence images, which may hinder the accurate determination of the plastic zone during this phase. As illustrated in Figure 12c,d, the assessed values for the three *a*/*W* values generally located within the 20% error band during the middle and late stages of the uniaxial tensile test. However, the estimation errors observed during the initial stretching stage slightly exceed the 20% range, which is consistent with the situation and corresponding reasons reflected in Figure 12a,b. Nevertheless, the findings demonstrate that the plastic zone can be characterized using characteristic values extracted from fluorescence images.

Unlike conventional detection techniques such as acoustic emission or DIC, the signals generated by MCL materials can be easily recognized by the naked eye and do not require complex equipment for capture and interpretation. Moreover, the MCL response is triggered by UV light, which can last a long period. Therefore, the simple CCD camera can effectively capture the dynamically changing fluorescence response produced by TPE-4N sensing film in real time. Additionally, operators can directly employ various programming languages, such as MATLAB or Python, or access open-source computer vision libraries through these languages to efficiently conduct post-processing of fluorescence images and perform in-depth mining of image information. The process of acquiring and analyzing the fluorescence response described above can be applied to small and irregular fluorescent areas such as the plastic zone near the crack tip. Therefore, the present method can be applied to large-scale and complex structures in service.

However, there are still many challenges for applications in the real engineering industry. It is reported that the mechanoresponsive luminescence response on stainless steel is not the same compared with that on aluminum alloy [30]. Thus, it means that the relationship between mechanoresponsive luminescence response features and plastic zone parameters should be calibrated when the substrate is changed. Moreover, fluorescence intensity is highly dependent on the measurement device. Namely, the camera exposure time, UV lamp power, and incidence angle may bring difficulty for quantitative analysis of fluorescence intensity. Therefore, the standardization of measurement devices is highly needed. Furthermore, the TPE-4N sensing film is a pure organic molecular film, thus the durability of this MCL sensing film in high temperature and humidity environments should be investigated before the real engineering applications.

## 5. Conclusions

In this study, the mechanochromic luminescent material was coated on the CT specimen surface as a sensing film. The evolution of the fluorescence response near the crack tip was investigated under various *a*/*W* values under uniaxial tension. The characterization parameters of fluorescence response were thoroughly mined, confirming a significant correlation between the characteristic values of fluorescence images and the plastic zone near the crack tip. Furthermore, this work has established a forward model that can quickly and accurately determine the maximum equivalent strain at the crack tip and the plastic zone size based on effective characterization parameters of fluorescence images and experimental variables. The following conclusions can be drawn:

(1) The local deformation near the crack tip results in the dynamically visible green fluorescence emission of the TPE-4N sensing film. The fluorescence distribution obtained using the MRL-based method agrees well with the equivalent strain distribution measured using the DIC method.
(2) The preprocessing of the fluorescence images results in normalized, denoised grayscale images of the crack tip. An enhanced watershed image segmentation algorithm is employed to segment out the ROI near the crack tip, yielding two simple characteristic values that describe the area and brightness of this region. The grayscale histogram and the GLCM, derived from the normalized denoised grayscale image, are analyzed to extract first-order and second-order characteristic values that quantify the texture features of the fluorescence image. These three types of characteristic values serve as potential characterization parameters for fluorescence response in the establishment of the MRL-based forward model of the plastic zone.
(3) The characteristic values derived from fluorescence images exhibit significant correlations with the maximum equivalent strain at the crack tip and the plastic zone size obtained through the DIC method. The plastic zone parameters determined using the MRL-based method agree well with the results measured using the DIC method. This indicates that the plastic zone near the crack tip can be effectively analyzed by capturing loading conditions and fluorescence response.

## Figures and Tables

**Table 1:** Results of Spearman rank correlation analysis for simple characteristic values.

Characteristic Value	Area (mm^2^)	Average Fluorescence Intensity
Maximum equivalent strain (%)	0.648 **	0.415 **
Plastic zone size (mm)	0.587 **	0.390 **

** *p* < 0.01.

**Table 2:** Results of Spearman rank correlation analysis for first-order characteristic values.

Characteristic Value	Mean	Std. Dev.	Smoothness	Uniformity	Entropy	Skewness
Maximum equivalent strain (%)	0.668 **	0.687 **	−0.687 **	−0.524 **	−0.285 **	0.658 **
Plastic zone size (mm)	0.646 **	0.638 **	−0.638 **	−0.522 **	−0.283 **	0.603 **

** *p* < 0.01.

**Table 3:** Results of Spearman rank correlation analysis for second-order characteristic values.

Characteristic Value	Contrast	Dissimilarity	Homogeneity	ASM	Energy	Correlation
Maximum equivalent strain (%)	0.634 **	0.613 **	−0.550 **	−0.464 **	−0.464 **	0.637 **
Plastic zone size (mm)	0.605 **	0.603 **	−0.549 **	−0.467 **	−0.467 **	0.569 **

** *p* < 0.01.

**Table 4:** Error analysis of multiple linear regression assessment.

Plastic Zone Parameter	R^2^	MAE	MSE	RMSE
*ε_e_* ^1^ from MLR (%)	0.91568	0.00227	8.01924 × 10^−6^	0.00283
*r* ^2^ from MLR (mm)	0.86262	0.04986	0.00512	0.07158

^1^ *ε_e_* is the maximum equivalent strain at the crack tip, ^2^ *r* is the plastic zone size.

## Data Availability

The original contributions presented in the study are included in the article, further inquiries can be directed to the corresponding author.

## Abbreviations

The following abbreviations are used in this manuscript:

MCL	Mechanochromic luminescence
AIE	Aggregation-induced emission
DIC	Digital image correlation
MRL	Mechanoresponsive luminescence
ML	Mechanoluminescence
TPE-4N	1,1,2,2-tetrakis (4-nitrophenyl) ethene
NDT	Non-destructive testing
MFL	Magnetic-flux-leakage
FEM	Finite element method
GLCM	Gray Level Co-occurrence Matrix
CT	Compact tensile
CCD	Charge-coupled device
UV	Ultraviolet
LED	Light-emitting diode
SNR	Signal-to-noise ratio
ROI	The region of interest
ASM	Angular Second Moment
K-S	Kolmogorov–Smirnov
S-W	Shapiro–Wilk
MLR	Multiple linear regression
R^2^	Coefficients of determination
MAE	Mean absolute error
MSE	Mean square error
RMSE	Root-mean-square error

## Appendix A

**Table A1:** K-S and S-W normality test for simple characteristic values.

Normality Test Method	Area (mm^2^)	Average Fluorescence Intensity
*p*-value of K-S test	8 × 10^−17^	0.01146
*p*-value of S-W test	2 × 10^−20^	0.00007

Data follow a normal distribution when *p* > 0.05.

**Table A2:** K-S and S-W normality test for first-order characteristic values.

Normality Test Method	Mean	Std. Dev.	Smoothness	Uniformity	Entropy	Skewness
*p*-value of K-S test	9 × 10^−10^	4 × 10^−8^	2 × 10^−25^	2 × 10^−10^	5 × 10^−8^	5 × 10^−42^
*p*-value of S-W test	1 × 10^−11^	2 × 10^−9^	6 × 10^−19^	2 × 10^−10^	7 × 10^−8^	2 × 10^−22^

Data follow a normal distribution when *p* > 0.05.

**Table A3:** K-S and S-W normality test for second-order characteristic values.

Normality Test Method	Contrast	Dissimilarity	Homogeneity	ASM	Energy	Correlation
*p*-value of K-S test	2 × 10^−9^	0.00022	0.00712	3 × 10^−10^	0.00095	5 × 10^−13^
*p*-value of S-W test	2 × 10^−11^	2 × 10^−7^	0.00001	2 × 10^−12^	10 × 10^−8^	4 × 10^−14^

Data follow a normal distribution when *p* > 0.05.
